# Physician Perception of the Importance of Medical Genetics and Genomics in Medical Education and Clinical Practice

**DOI:** 10.1080/10872981.2022.2143920

**Published:** 2022-11-08

**Authors:** Emilie Logan French, Leena Kader, Erin E. Young, Joseph D. Fontes

**Affiliations:** a Department of Anatomy and Cell Biology, University of Kansas School of Medicine, Kansas City, KS, USA; b Department of Anesthesiology, University of Kansas School of Medicine, Kansas City, KS, USA; c Department of Biochemistry and Molecular Biology, University of Kansas School of Medicine, Kansas City, KS, USA

**Keywords:** Genetics, genomics, medical education, physician perception

## Abstract

The importance of medical genetics and genomics in medical practice has grown proportionally with scientific advances in these areas.

**Purpose:**

The objective of this study was to determine physician perceptions regarding the importance of and comfort with the use of medical genetics and genomics in medical education and practice, as well as physician expectations for medical trainees.

**Methods:**

A retrospective survey was sent to physicians employed by a health system associated with a public medical school to assess their perceived training in medical genetics and genomics and their comfort level with ordering genetic testing.

**Methods:**

Despite reporting formal genetics training in medical schools, clinicians’ comfort with and knowledge in this content area does not meet personal expectations of competency. Though physicians report some discomfort with the use of medical genetics and genomics, the majority also believe that its impact on practice will increase in the next five years. Survey recipients were also asked about their expectations for preparation in the same domains for medical students and incoming residents. The surveyed physicians expect a high level of competency for medical students and incoming residents.

**Methods:**

Our study revealed that practicing physicians feel current medical curricula do not produce physicians with the necessary competency in medical genetics and genomics. This is despite physicians’ perceived importance of this domain in medical practice. Our findings suggest a need for re-evaluation of medical genetics and genomics education at all levels of training.

## Introduction

Knowledge about the human genome and opportunities to use medical genetics and genomics in medical practice has expanded in combination over the last couple of decades [1]. Despite these advancements, various barriers remain to the integration of medical genetics and genomics into routine medical practice. These barriers include concerns about ethical issues, patient anxiety, insurance discrimination, access to genetics expertise, and time for practitioners to keep abreast of advances [2,3]. Such obstacles may reduce the application of medical genetics and genomics in the clinic with a loss of benefit to patients [4–6]. The risk of lost benefits will continue to grow as new clinical genetic and genomic tests enter the market each year [7–9].

Virtually all medical specialties are impacted by genetics and genomics. Ideally, physicians in all types of practice should be comfortable applying medical genetics and genomics to patient care [8,10]. About 10% of Americans have a rare disease, and up to 80% of these rare diseases have a genetic basis, making a significant portion of the American public potential beneficiaries of practitioners fluent in medical genetics and genomics [11]. Beyond rare diseases, personalized medicine – the application of medical genetics and genomics to assessing risk, diagnosis, and management of common conditions – has the potential to dramatically change medical practice and improve patient outcomes [8,12,13]. Several recent reports have highlighted the need for training current and future physicians in the application of medical genetics and genomics to expand personalized medicine approaches; this is especially acute given the relatively small number of physicians board certified in medical genetics [8,9,14,15]. For such training to change practice successfully, physicians and trainees must believe that this education is necessary to be fully competent practitioners and that it has the potential to improve the health of their patients and communities [10,16,17].

Given the barriers to the application of medical genetics and genomics in clinical practice, we undertook this study to better understand physician utilization and attitudes regarding these fields in medical education and practice. In addition, we wanted insight into medical school faculty physician comfort with the same and how this impacted their practice and attitudes towards trainee preparation. Such findings can inform decision-making and design regarding undergraduate and graduate medical curricular reform and the potential need for increased offerings in continuing medical education surrounding medical genetics and genomics.

## Materials and methods

### Research Design

This study was a retrospective survey sent to physicians in the following specialties at the University of Kansas Health System in the Kansas City, Kansas metro area: General Surgery, Obstetrics and Gynecology, Otolaryngology, Internal Medicine, Hospital Medicine, Family Medicine, Cardiology, and Pediatrics. All physicians are faculty members of the University of Kansas School of Medicine.

### Participant Selection and Recruitment

Every physician from each listed specialty at https://findadoctor.kansashealthsystem.com/ (accessed July 1^st^, 2020) was included in the recruitment email.

The University of Kansas Medical Center (KUMC) directories were used to identify subjects and their email addresses. A recruitment email was sent to each physician in July 2020. A reminder was sent 11 days later, and a final call for responses was sent two months after the first email. The survey was sent to a total of 262 physicians in the following specialties: Pediatrics (24), Internal Medicine (42), Hospital Medicine (73), Family Medicine (38), OBGYN (14), General Surgery (4), ENT (25) and Cardiology (42).

### Measurement Tools and Data Collection

Data were collected from a survey adapted from Chow-White, et al., and Haga et al. [18,19]. Portions of explanations for types of genetic tests were essentially used verbatim from Chow-White, et al., and Haga et al. [18,19]. The survey and study design were approved by the Institutional Review Board of the University of Kansas Medical Center and conducted according to the ethical principles outlined in the Belmont Report, specifically, the three key concepts: respect for persons, beneficence, and justice. The physicians filled out the survey, which was expected to take no longer than 10 minutes. No personal identifying information was collected.

### Statistical Analyses

All data were exported to Statistical Package for the Social Sciences (SPSS) v26 (IBM, Inc., Armonk, NY, USA) for subsequent analyses. Count data were analyzed using the X^2^ test (p < 0.05) while self-report Likert scale measures were analyzed using t-tests and/or Analysis of Variance (ANOVA) as appropriate (p < 0.05). All graphs were produced using GraphPad Prism v9 (Dotmatics, Boston, MA, USA).

## Results

 Of the 262 physicians who were sent the survey, 65 responded for a 25% response rate. Most of the respondents practice in an academic medical center setting with a small number of physicians in community practice. We had respondents from all queried departments (Pediatrics, Obstetrics/Gynecology, Family Medicine, Internal Medicine, Hospitalists, Otolaryngology, and Cardiology) except General Surgery (Table 1).

**Table 1.** Demographic information for population surveyed, Kansas, USA, July 2020.

Number of Respondents 65 (100%)	
**Age (years)**	
25–34	4 (6.2%)
35–44	27 (41.5%)
45–54	9 (13.8%)
55–64	16 (24.6%)
65+	9 (13.8%)
**Medical School Graduation Year**	
Prior to 1980	7 (10.8%)
1981–1990	14 (21.5%)
1991–2000	13 (20.0%)
2001–2010	21 (32.3%)
2011–2020	10 (15.4%)
**Primary Clinical Setting**	
Acad. Med. Center	56 (86.2%)
Community	5 (7.7%)
Both	4 (6.2%)
**Practice Specialty**	
Pediatrics	7 (10.8%)
Obstetrics/Gyn.	6 (9.2%)
Family Medicine	11 (16.9%)
Internal Medicine	19 (29.2%)
Hospital Medicine	2 (3.1%)
General Surgery	0 (0%)
Otolaryngology	9 (13.8%)
Cardiology	11 (16.9%)

**Figure 1.**
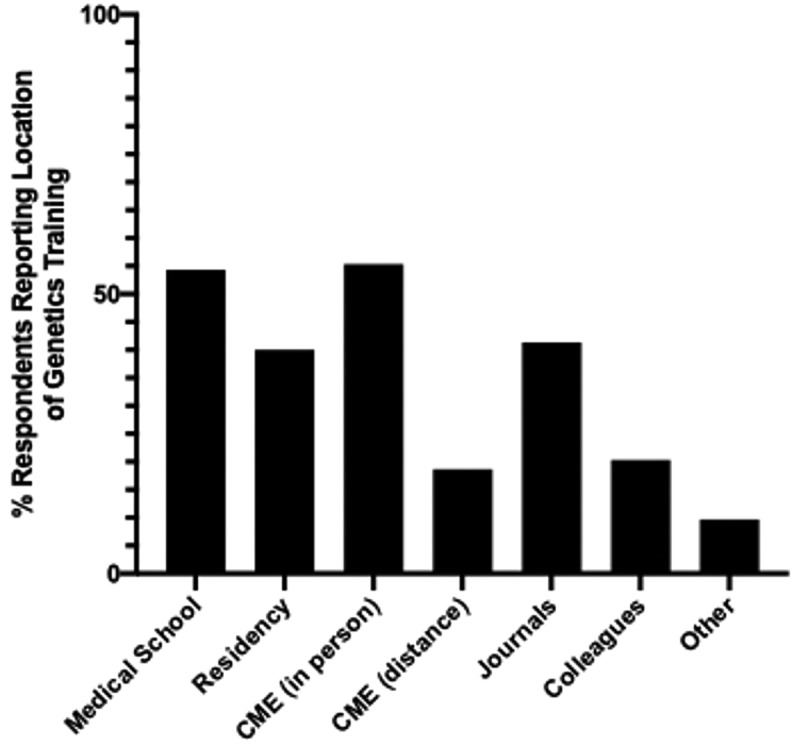
Sources of genetics training across all respondents. Most common sources of genetics training included medical school (n = 42; 65%) and continuing medical education (CME) in person (n = 39; 60%) followed by journals (n = 36; 55%) and residency training (n = 31; 48%). Those who marked ‘other’ had a variety of responses (23andMe, blogs, BioStar course, genomics course work, internet, informatics trade shows, personal experience with genetic conditions, and undergraduate education).

**Figure 2.**
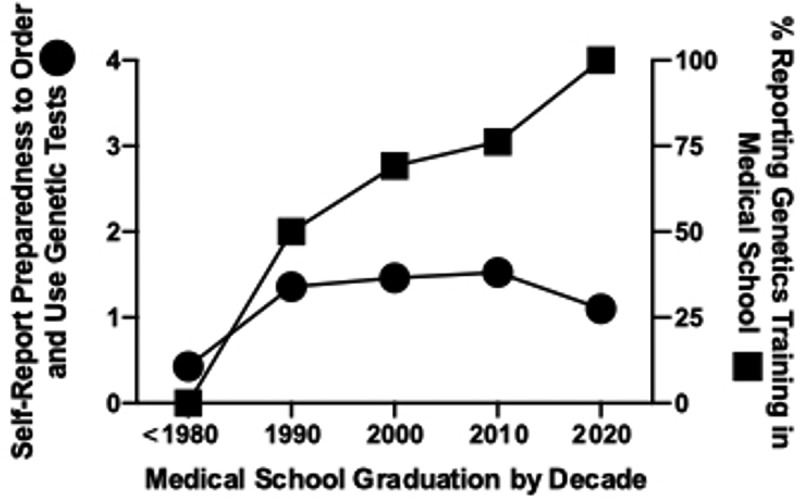
Number of respondents who self-reported preparedness to order and use genetic tests (left axis; see Table 1 for number of respondents in each graduation decade) and percent of respondents reporting genetics/genomics content in medical school (right axis), as a function of medical school graduation by decade.

We first wanted to determine the source and extent of genetics education and the impact of recency of genetics training on self-reported preparedness to order and use genetic tests. Among our respondents, the two most common sources of genetics training reported were medical school and continuing medical education (CME) in person, followed by residency and journals (Figure 1). We suspected that more recent medical graduates might have received greater genetics education overall, corresponding with the expansion of medical genetics and genomics knowledge; in general, this was supported by our findings (Figure 2). Notably, none of those who graduated before 1980 (n = 14; Table 1) reported genetics training in medical school. This contrasts with 100% of graduates from the past decade reporting genetics training in medical school (Figure 2). However, except for graduates before 1980, there was no correlation between the percentage reporting medical school training in genetics and the respondents’ comfort with ordering and using genetic tests as physicians (Figure 2).

To parse the impact of genetic training on comfort with the application of genetics in medical practice, respondents were asked to indicate whether they agree or disagree with the statement ‘I feel that my genetics training adequately trained me to order and use genetic tests’. Responses clustered around ‘somewhat disagree,’ with 63% of respondents answering either strongly disagree (23%) or somewhat disagree (40%) (n = 41). 15% of providers somewhat agreed that their genetics training adequately prepared them to appropriately use and order genetic tests (n = 10). Strikingly, no respondents marked ‘strongly agree’ to this question.

We anticipated that physicians who felt adequately prepared by their medical genetics and genomics training might also have greater comfort in ordering specific genetic tests. In general, this was true (Table 2). Looking solely at physicians who agreed their training in genetics adequately prepared them (Prepared, n = 10), the majority also felt well informed about genetic testing in general (n = 7, 70%). Additionally, most of the adequately prepared group felt comfortable ordering a genetic test to diagnose disease (n = 8, 80%), or for disease susceptibility (n = 6, 60%; Table 2). Of note, the adequately prepared group also reported good access to genetics expertise (n = 8, 80%; Table 2). An exception to this overall trend was mixed comfort with pharmacogenetic testing for risk of adverse events/outcomes (30% agreed, 40% disagreed).

**Table 2.** Respondent physicians who reported comfort with using medical genetics and genomics in the practice setting by category of application and who reported access to genetics expertise, grouped by whether they felt their genetics training was adequate (prepared vs. unprepared).

		Agreed (%)	Neutral (%)	Disagreed (%)
Well Informed in General	Prepared	70	20	10
	Not Prepared	22	29	49
Comfortable Ordering a Test for Diagnosis	Prepared	80	10	10
	Not Prepared	34	22	44
Ordering Tests for Disease Susceptibility	Prepared	60	20	20
	Not Prepared	37	17	46
Pharmacogenetics	Prepared	30	30	40
	Not Prepared	17	15	68
Access to Expertise	Prepared	80	20	0
	Not Prepared	27	17	56

In contrast, we expected that physicians who did not feel prepared by their genetics training would have lower comfort levels for ordering various genetic tests (‘not prepared’, n = 41), and this holds across all testing modalities investigated: diagnostic tests, pharmacogenetic tests, and susceptibility tests (Table 2). As expected, physicians who did not feel prepared by their training also felt less informed in general about medical genetics and genomics. Importantly, these same physicians self-reported lower levels of access to expertise.

**Figure 3.**
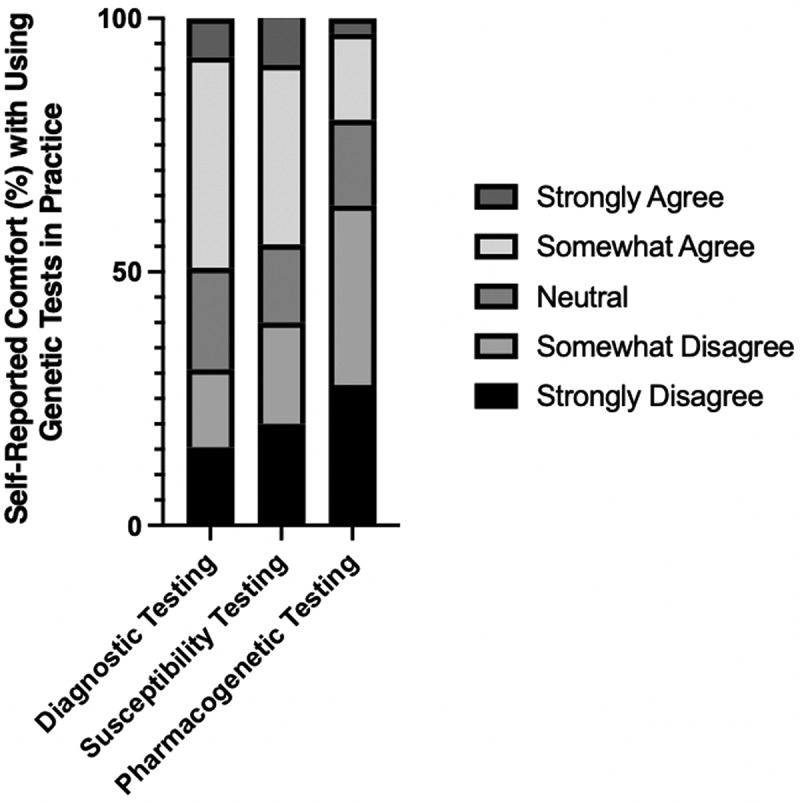
Respondent physicians’ reported comfort to order and use diagnostic, susceptibility, and pharmacogenetic testing on a Likert Scale from ‘Strongly Disagree’ to ‘Strongly Agree.’.

We also queried respondents on their comfort level with ordering and using genetic tests in practice. On a five-point Likert scale, the average response regarding preparedness to use multiple types of genetic testing clustered around ‘Neutral’ (for diagnostic and disease susceptibility testing) and ‘Somewhat disagree’ (for pharmacogenetic testing; Figure 3).

**Figure 4.**
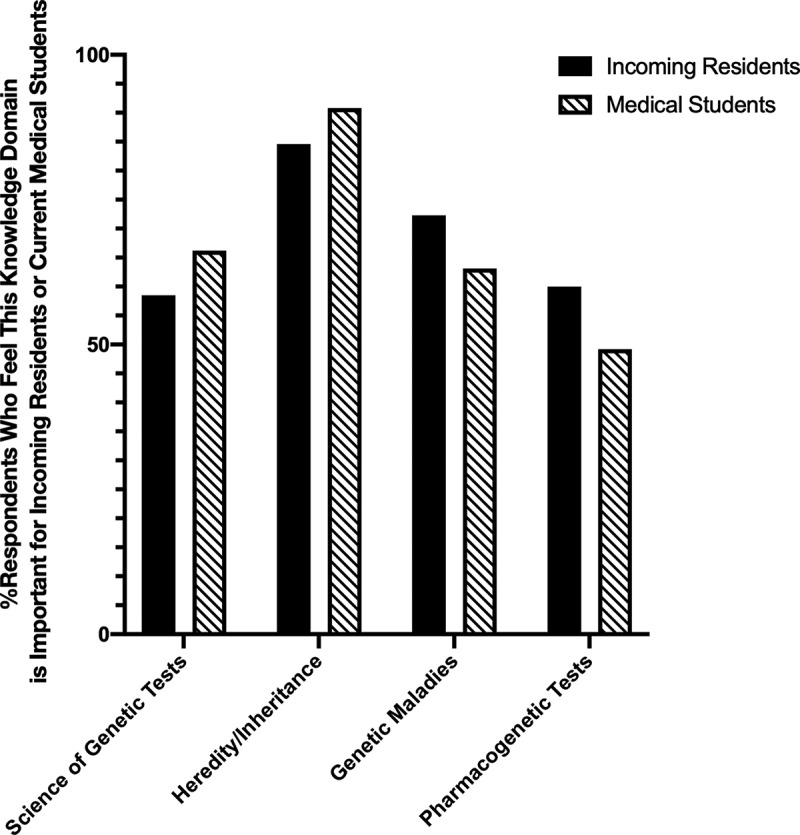
Respondent physician’s belief in the importance of various domains of medical genetics and genomics for an incoming resident and current medical student.

While physician responses regarding their comfort with genomics knowledge and genetic testing revealed a relatively low comfort level, they held generally high expectations for medical students and incoming residents. A majority of all respondents reported that three of four separate genetic knowledge domains were important for medical students and four of four domains for incoming residents (Figure 4). The overall high expectations for genetics training in trainees are consistent with most respondents reporting that impact of genetics and genomics on multiple aspects of medical practice to be consistent or increase over the next five years (Supplemental Figure 1).

## Discussion

Despite perceived importance, supported by their expectations for trainees, we found that most physicians in our survey reported relatively low comfort with applying medical genetics and genomics in the clinical setting. These feelings might be linked to their perceived poor preparation during training, which highlights the importance of effective medical genetics and genomics education in medical school through CME. Compounding the sense of being unprepared for the use of medical genetics and genomics in medicine, many in our survey also reported limited access to genetics expertise. This perception of limited access to expertise was especially evident in physicians who did not feel prepared by their genetic training, but as all physicians surveyed work at the same academic center, they theoretically have access to the same expertise. This suggests that a large proportion of the physicians in our survey feel both underequipped to use medical genetics and genomics and unable to access genetic specialists as needed. This may indicate that a perceived lack of training contributes to a perceived, but not actual, lack of access to genetics expertise. Our findings regarding the lack of physician comfort with medical genetics and genomics are in accordance with several reports published over the past two decades [3,20–22].

The expansion of genetics content in medical curricula has generally tracked with advances in human medical genetics and genomics, based upon physician self-evaluation of competency and preparedness. Despite this expansion of genetics content in undergraduate medical curricula over time, we found that training did not engender confidence in physicians regarding the application of medical genetics and genomics in practice. This suggests a need to reform current medical genetics and genomics education to an orientation of application, with outcomes focused on clinical utility. As medical school and in-person CME were the two most common locations of genetics training for physicians, these two settings may provide the greatest benefit for educational reform. Focus on these settings will not only capture future physicians but ensure those currently in practice increase their comfort with applying clinical tools involving medical genetics and genomics.

Most medical genetics and genomics education at U.S. medical schools is found in the pre-clerkship years and is often limited to general genetic concepts rather than the application of that knowledge [23,24]. Indeed, the genetic knowledge required for USMLE licensing exams tracks with trends in curricula, focusing on general principles and not the application to practice: this is occurring in the context of reduced expectations of genetic knowledge over time [25]. Expansion and integration of medical genetics and genomics throughout clinical training may be a missed opportunity, as it is well established that longitudinal exposure in authentic settings leads to more effective learning. Ideally, exposure to applied medical genetics and genomics in medical school would be followed by structured education throughout post-graduate training [14]. However, even though many studies have recognized the need for improved medical genetics and genomics education, content in medical education programs is already very dense, so careful thought must be given to potential revisions [5,14].

Physicians who reported feeling prepared by their genetic training, not surprisingly, felt more comfortable with all types of genetic tests. This suggests that better education can lead to the desired outcome: physicians who understand and use genetic tests in everyday practice. However, physicians who reported feeling prepared and those who reported feeling unprepared both cited a lack of understanding and comfort with pharmacogenetics tests specifically. This points to an opportunity for expanded education in this area, with significant potential to improve drug safety and patient outcomes [26].

Access to genetics expertise might be expected to improve physician comfort with medical genetics and genomics in the clinic. However, our data suggested that physicians who did not feel prepared by their genetics training perceived less access to genetics expertise, despite theoretically having the same access to expertise as other physicians in practice at this academic center.

Despite a self-perceived lack of comfort with the topic, physicians had high expectations for medical students and incoming residents for medical genetics and genomics competency in multiple domains. The physicians’ belief in the expanding importance of medical genetics and genomics in clinical practice over the next five years may partially explain their high expectations for trainees. Interestingly, reported expectations for knowledge about pharmacogenetic tests in medical students were lower, matching up with physicians’ own, reported inferior level of knowledge about the same. Similar findings were not seen for expectations for incoming residents, which is somewhat incongruous as incoming residents were medical students just one year prior.

## Limitations

This survey was conducted with physicians from KU hospital, which is a single academic medical center, and our final response rate was modest at 25%. As there was a low number of respondents for many specialties (and no respondents for General Surgery) we were unable to conduct specialty-specific analyses which was our original plan.

Application of medical genetics and genomics has been established as an important aspect of clinical medicine and this importance will continue to expand as discoveries are translated to the clinic. The results of our survey point to a mismatch between current medical education and the attainment of optimal physician competencies in medical genetics and genomics. Reform of medical school curricula and CME might best address this mismatch by expanding training in the application of medical genetics and genomics to clinical problem-solving, rather than basic genetic concepts. Longitudinal exposure to this education will likely lead to better application and thus benefits to patients. For the self-perceived, unprepared physicians, education needs to be aimed not only at expanding knowledge but increasing awareness of accessibility to genetics expertise. Additional research aimed at eliciting medical specialty-specific focused curricula in medical genetics and genomics, as well as the best way to deliver it to practicing physicians, is needed to inform CME practices. Our understanding of medical genetics and genomics, and therefore its usefulness, is only going to continue to increase. Therefore, it is important that both trainees and physicians in practice be trained to assimilate and apply continually expanding medical genetics and genomics knowledge for the benefit of their patients.

## Supplementary Material

Supplemental Material
