# Supplementary material for: Physician Perception of the Importance of Medical Genetics and Genomics in Medical Education and Clinical Practice
Source: Med Educ Online. 2022 Nov 8;28(1):2143920. doi: 10.1080/10872981.2022.2143920 (PMC9648379; doi:10.1080/10872981.2022.2143920)
Supplement: Supplemental Material [file ZMEO_A_2143920_SM9856.zip › Suppl. Files/Supplementary figure legend.docx]

**Supplemental Figure 1.** Respondent physician’s belief in the impact of medical genetics and genomics on domains of medical practice in the next 5 years, by year of graduation from medical school.
