# Supplementary figures and images for: Physician Perception of the Importance of Medical Genetics and Genomics in Medical Education and Clinical Practice
Source: Med Educ Online. 2022 Nov 8;28(1):2143920. doi: 10.1080/10872981.2022.2143920 (PMC9648379; doi:10.1080/10872981.2022.2143920)

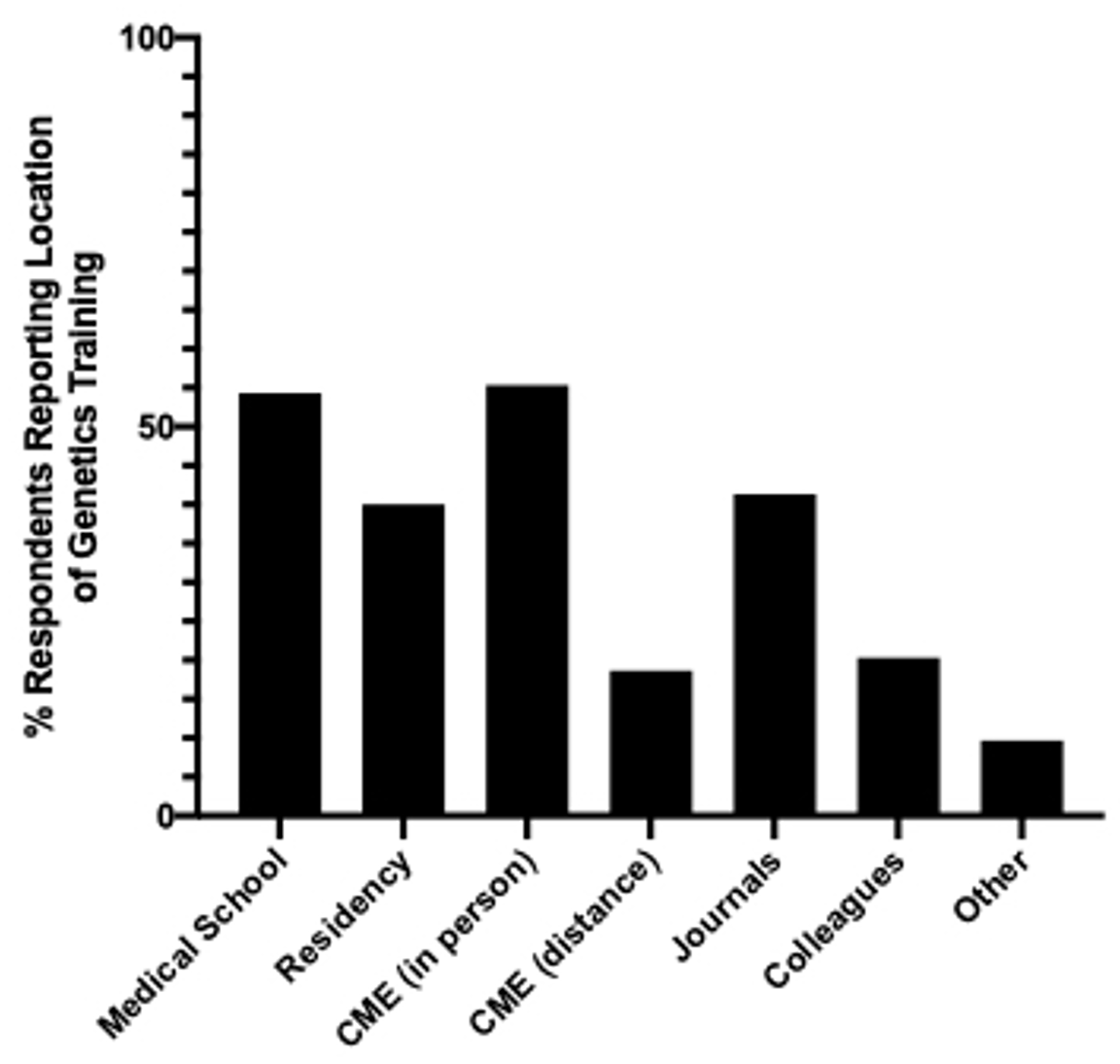

Supplement: Supplemental Material [file ZMEO_A_2143920_SM9856.zip › Suppl. Files/Suppl Figure 1.png]
